# Arsenic immunotoxicity: a review

**DOI:** 10.1186/1476-069X-12-73

**Published:** 2013-09-02

**Authors:** Nygerma L Dangleben, Christine F Skibola, Martyn T Smith

**Affiliations:** 1 Division of Environmental Health Sciences, School of Public Health, University of California, Berkeley, CA 94720, USA; 2 Department of Epidemiology, School of Public Health, University of Alabama, Birmingham, AL 35294, USA

**Keywords:** Arsenic, Immune system, Immunotoxicity, Immunocompromised, Immunosuppression

## Abstract

Exposure to arsenic (As) is a global public health problem because of its association with various cancers and numerous other pathological effects, and millions of people worldwide are exposed to As on a regular basis. Increasing lines of evidence indicate that As may adversely affect the immune system, but its specific effects on immune function are poorly understood. Therefore, we conducted a literature search of non-cancer immune-related effects associated with As exposure and summarized the known immunotoxicological effects of As in humans, animals and *in vitro* models. Overall, the data show that chronic exposure to As has the potential to impair vital immune responses which could lead to increased risk of infections and chronic diseases, including various cancers. Although animal and *in vitro* models provide some insight into potential mechanisms of the As-related immunotoxicity observed in human populations, further investigation, particularly in humans, is needed to better understand the relationship between As exposure and the development of disease.

## Background

Exposure to arsenic (As) is a global public health concern because As is widely distributed and associated with numerous adverse effects. As is a well-established cause of skin, lung and bladder cancers in humans [1], and is associated with skin lesions, diabetes, cardiovascular disease and other disorders [1-3]. Well over 100 million people worldwide are exposed to As, particularly through ingestion of contaminated food and water in countries such as India, Bangladesh, Taiwan, Chile, and the United States [1,4]. Exposures also occur through inhalation, especially in agricultural and industrial settings [4].

Inorganic As exists in the environment as arsenite (As^III^) or arsenate (As^V^) and is metabolized in humans via conversion of As^V^ to As^III^ with subsequent methylation to mono- and di-methylated arsenicals (MMA and DMA, respectively) [5]. MMA^III^ is considered the most toxic arsenical *in vitro*[6-9] and individuals who excrete a higher proportion of ingested As as urinary MMA have increased risks of As-associated cancers [10,11], suggesting a key role for MMA in As toxicity. Proposed mechanisms of toxicity include oxidative stress, inhibition of DNA repair, chromosomal aberrations, micronuclei formation, induction of apoptosis, modification of cellular signaling via altered activation, expression and DNA binding activity of transcription factors, epigenetic modifications resulting in aberrant gene expression, and altered phenotype of stem cell populations [12-16]. As toxicity is thus complex and multifaceted, but is not yet well understood.

Although extensive research has focused on investigating As carcinogenicity, growing evidence indicates that As also has deleterious effects on the immune system [17,18]. This may potentially play a role in As carcinogenesis of various tissues through reduced immune surveillance. However, the specific effects of As on immune function remain poorly understood. Therefore, we considered that further investigation of As immunotoxicity is warranted and conducted a PubMed search of As exposure and non-cancer immune-related effects through October 2012. Here, we summarize the known toxicological effects of As on immune function in humans, laboratory animals and *in vitro* models, and identify possible future research directions to help close the gaps in knowledge.

## Epidemiological findings

### Effects in adults

#### Gene expression

Microarray-based assays are widely used for identifying differentially expressed genes in investigations of As carcinogenicity. However, a limited number of reported epidemiological studies have employed this powerful method to investigate As toxicity in immune cells from otherwise healthy persons. A microarray-based genome-wide expression study of peripheral blood mononuclear cells (PBMC) from 21 subjects in New Hampshire whose drinking-water As averaged 0.7 μg/L (range 0.007–5.3 μg/L, *n* = 10) and 32 μg/L (range 10.4-74.7 μg/L, *n* = 11) showed significant differences between exposure groups in transcripts with functions in T-cell receptor signaling, cell cycle regulation and apoptosis, and most strikingly defense and immune response [19]. Notably, higher As exposure was associated with increased expression of killer cell immunoglobulin-like receptors that inhibit natural killer cell-mediated cytotoxicity, as well as decreased expression of MHC class II molecules, *HLA*-*DQB1*, *HLA*-*DPA1*, and *HLA*-*DRB1*; defense response genes, *CD69*, *HSPA9B* and *MALT1*; and inflammatory genes, *IL2RA* and *IL1B*[19]. Exposure was determined by levels of drinking-water As combined with urinary or toenail As as internal markers of exposure, and control and exposed subjects were matched for age, sex and smoking status.

Down-regulated *IL1B* was also identified in a microarray study of PBMC from an As-exposed Bangladeshi population with (*n* = 11) and without skin lesions (*n* = 5) [20]. An overall suppression of 467/468 differentially expressed genes was observed. These findings contradict those from a microarray analysis of PBMC from 24 individuals in Taiwan with low (0–4.32 μg/L), intermediate (4.64–9 μg/L), and high (9.6–46.5 μg/L) blood As levels [21]. Among 62/708 significantly altered genes were several inflammatory molecules that were up-regulated, including *IL1B*, *IL6*, *CCL2* and *CD14*, indicating that prolonged exposure may induce ongoing inflammation that could contribute to As-associated disease [21].

More recently, a cDNA microarray study of PBMC from 10 individuals in Mexico having urinary As levels between 117.23 and 435.12 mg/g creatinine (*n* = 5) revealed significant differences in expression of apoptosis- and inflammation-related genes compared to unexposed subjects (*n* = 5) [22]. Exposure was associated with down-regulated inflammatory genes, including *TNF*, *IL11*, *IL10RB*, *CCR1*, and *CXCL2*[22], which is in stark contrast with up-regulated inflammatory genes reported in chronically-exposed persons in Taiwan [21]. However, the finding of decreased *TNF* concurs with data from the Bangladeshi study [20]. Some apoptosis-related genes were significantly up-regulated, including *BCL2L1* and *CASP2*, whereas others, namely *TRAIL* and *FASLG*, were suppressed [22]. Such contradictory results could be influenced by differences in exposures, sampling, methodology, population genetics and/or environmental factors. For instance, the Mexican study [22] analyzed individual RNA samples from each subject, whereas the Taiwanese study [21] used pooled samples. Additionally, the small number of participants may not be large enough to draw definitive conclusions.

One caveat is that changes in expression may not necessarily be viewed as toxic responses; some changes may occur following chemical exposure as adaptive responses, e.g. acquired resistance to acute toxicity, over time [23]. However, changes reported at the gene and/or protein level are provided to illustrate possible effects resulting from As exposure that may alter cellular function and ultimately the immune system’s ability to defend the host.

#### Lymphocyte activation

Impaired T-cell activation and functional responses have been observed in As-exposed persons. Analysis of 11 exposed and 13 control individuals in Mexico indicated that chronic exposure significantly decreased proliferation of mitogen-stimulated lymphocytes [24], which is supported by a later study identifying delayed cell cycle progression from S- to M-phase in chronically-exposed persons whose drinking-water levels averaged 412 μg As/L compared with persons consuming water averaging 37.2 μg As/L [25]. Similarly, a cross-sectional study in West Bengal, India of 18 controls and 20 As-exposed individuals with skin lesions found significant reductions in lymphoproliferation and Th1/Th2 secretion of IL-2, IL-4, IL-5, IL-10, IFN-γ and TNF-α in As-exposed compared with unexposed individuals [26]. In contrast, lymphocytes from Chilean copper smelter workers exposed to As-contaminated air (*n* = 40) displayed higher proliferation rates than those of As-unexposed individuals from the same region (*n* = 32) [27]. While reasons for the observed differential effects on T-cell proliferation remain unclear, it is plausible that differences in population genetics, metabolism, and/or exposure could be involved. The study also found that urinary As levels positively correlated with oxidative stress markers serum superoxide dismutase (SOD) and lymphocyte malondialdehyde (MDA), and negatively correlated with serum vitamin E levels, suggesting that chronic exposure induces lymphocyte oxidative damage [27].

#### Lymphocyte subpopulations

Increasing evidence indicates that As affects numerous immune cell subpopulations. Subjects exhibiting dermatological symptoms (*n* = 30) from exposure to > 100 μg As/L for > 10 years in Eastern India demonstrated significantly increased eosinophil numbers and decreased monocyte counts compared to unexposed persons (*n* = 25) [28]. As also disrupts macrophage function: monocyte-derived macrophages from As-exposed individuals with skin lesions (*n* = 70) demonstrated cell rounding and significantly reduced adhesion, nitric oxide anion (NO^-^) production and phagocytic capacity compared to macrophages from non-exposed persons (*n* = 64) [29]. Down-regulated F-actin and CD54 adhesion molecule, and altered Rho A-ROCK signaling likely contributed to impaired macrophage function.

Investigation of As influence on immune regulation revealed that in chronically-exposed but otherwise healthy individuals (*n* = 47), urinary As levels (range, 8.1 – 448 μg/g creatinine) significantly inversely correlated with the number and function of natural T regulatory (nTreg) lymphocytes but not other regulatory T-cells [30]. nTreg lymphocytes are CD25^+^Foxp3^+^ T-cells that constitute approximately 10% of circulating CD4^+^ T-cells and play a critical role in immune homeostasis by suppressing immune response [31]. Although increased apoptotic PBMC were evident in exposed subjects, no significant correlation was found with urinary As levels, suggesting that the effect on nTreg cells was not mediated by apoptosis induction [30]. Alternatively, this could be due to individual differences in As susceptibility. Exposure positively correlated with monocyte innate immune receptor complex TLR4/CD14 and TNF-α secretion [30], which may be causally associated with As effects on nTreg lymphocytes given their inhibitory effect on pro-inflammatory TNF-α release [32]. These results concur with previous findings that chronic human As exposure induces inflammation, including *CD14*[21].

#### Humoral immunity

Studies evaluating antibody levels in As-exposed individuals yield conflicting results. One study observed no changes in serum IgM, IgA or IgG in 47 adult male workers exposed to As in a coal-burning power plant compared to 27 workers from another plant in the same district whose As coal content was > 10 times lower [33]. It should be noted that exposure duration and internal As doses are unknown; thus, negative results could be due to acute or low-dose internal As levels. In contrast, Bangladeshi subjects (*n* = 125) chronically exposed to drinking-water As demonstrated significantly elevated serum IgA, IgG and IgE compared to unexposed persons [34]. IgG and IgE levels were significantly higher during initial stages of skin manifestations, and IgE continued to increase with prolonged exposure. Moreover, increased prevalence of respiratory complications including cough, chest sound, bronchitis and asthma were evident in exposed individuals, and mean serum IgE was higher in subjects with respiratory symptoms relative to exposed subjects without [34]. No effect on eosinophils was observed [34], in contrast with a reported As-associated increase in eosinophil numbers [28], suggesting that increased serum IgE may be due to direct inflammatory effects of As rather than allergic disease [34].

#### Pulmonary effects

Health outcomes of As immunosuppression are evidenced by increased prevalence of opportunistic infections such as tuberculosis and fungal and respiratory tract infections (RTI) [26,29,35]. A recent report from Chile revealed increased mortality from As-associated pulmonary tuberculosis [35]. Reports of As-associated pulmonary effects [26,29,34-36] support growing evidence indicating that long-term exposure increases risk of reduced lung function and non-malignant lung disease [36-41]. Moreover, epidemiological investigations provide compelling evidence that As increases the incidence of bronchiectasis [42,43], a pulmonary disease characterized by chronic infection, inflammation, irreversible bronchial damage, and respiratory failure [44,45]. Chronically-exposed subjects from West Bengal, India with As-related skin lesions (*n* = 108) demonstrated a 10-fold higher prevalence of bronchiectasis compared with subjects without lesions (*n* = 150) [42]. A later report from Chile indicated elevated mortality rates for bronchiectasis in adults aged 30–49 resulting from early-life As exposure; compared with controls, mortality rates for those with childhood and *in utero* exposure were 12- and 46-fold higher, respectively [43]. Also observed were 6- to 7-fold increases in lung cancer mortality rates resulting from early-life exposures. Studies on this As-exposed Chilean population indicate long latency patterns of increased lung, kidney and bladder cancer mortality continuing for > 25 years after exposures ended [46,47]. Overall, these reports indicate that As not only exerts severe respiratory effects, but that early-life exposures have pronounced long-term consequences that may include higher prevalence of and mortality from cancers of different tissues. Intriguingly, women appear to be somewhat protected from skin and respiratory manifestations [36,48], possibly due to sex hormone-related increased methylation capacity of As in women than in men [49].

#### HBD1 involvement

Interestingly, we previously reported in two As-exposed populations from Nevada and Chile a significant inverse correlation in men between urinary levels of As and antimicrobial peptide human β-defensin-1 (HBD1) [50]. Studies suggest a primary role for HBD1 against pulmonary pathogens relevant to bronchiectasis [44,45] and an association between HBD1 antimicrobial inactivation and recurrent airway infections in cystic fibrosis patients [51,52]. Further, observations from transgenic mice deficient in the mouse ortholog of HBD1 indicate that β-defensin-1 serves as an initial barrier to pulmonary bacterial colonization [53]. Given growing evidence that *DEFB1*, the gene encoding HBD1, is a putative tumor suppressor whose down-regulation may be involved in tumorigenesis of multiple tissues [54-62], it is tempting to speculate that HBD1 suppression may contribute to As-induced carcinogenesis or bronchiectasis. Although our ongoing studies demonstrate As-induced reductions in *DEFB1* mRNA and protein in human cell lines (unpublished data), confirmatory evidence of HBD1 inhibition is needed from other As-exposed populations. Thus, it remains to be determined whether HBD1 is suppressed in lungs of As-exposed individuals, and further investigations are needed to elucidate the role of down-regulated HBD1 in As immunotoxicity and carcinogenicity.

### Effects in children and infants

The fetus, infant and young child, each at critical stages in development, are particularly sensitive to stressors that could have short- and long-term effects. Yet, few epidemiological studies have investigated the influence of early-life As exposure on immunological outcomes in children and even fewer in newborns and infants. Evidence indicates that early-life As exposure may have consequences that manifest much later in adulthood [18,63], as evidenced by increased prevalence of and mortality from bronchiectasis and lung cancer in young adults [43]. Therefore, biomarkers indicative of future disease following early-life exposure could be evident in young subjects.

#### Induction of apoptosis

Indeed, studies of early-life As exposure have detected markers of immune dysfunction in infants and children. Studies of Mexican children aged 4–13 have reported higher incidences of apoptotic PBMC in As-exposed children relative to controls [64,65]. Although apoptosis is important in immune homeostasis, abnormal immune cell apoptosis can contribute to dysregulated immune function, which may result in immunodeficiency, autoimmune disease or malignant transformation [66]; thus, induced apoptosis may be important in As-mediated immunosuppression. The larger study of 40 children (high and low mean urinary As levels = 46.3 and 14.2 μg/g creatinine, respectively) found a significant positive association between As exposure and apoptotic PBMC [65]. However, despite elevated apoptotic PBMC in chronically-exposed children from the smaller study of 7 highly-exposed and 5 non-exposed children (mean urinary As levels = 143.9 and 24.8 μg/g creatinine, respectively), no significant correlation was observed between exposure and apoptotic cells [64], in agreement with a study on adults [30], possibly due to small sample size or individual differences in As susceptibility [64].

#### Lymphocyte activation

Consistent with findings from adults [24-26], significant reductions in PBMC IL-2 secretion and proliferation were observed in As-exposed children aged 6–10 (*n* = 90, mean urinary As levels of high- and low-exposure group = 194.9 and 29.3 μg/L, respectively) [67]. Also noteworthy were increased granulocyte-macrophage colony stimulating factor (GM-CSF) secretion and reduced CD4^+^ cell count and CD4/CD8 ratio without altered CD8^+^ cell proportion [67]. Because low CD4/CD8 is considered a surrogate marker of immunosuppression [68-70], the observed decrease in CD4/CD8 may be an early indicator of As-mediated immunosuppression. Furthermore, the increased GM-CSF secretion may indicate chronic inflammation given growing evidence of elevated GM-CSF levels in initiating/mediating chronic inflammation [71], and is consistent with a previous study of As-exposed adults demonstrating up-regulated inflammatory molecules [21].

#### ROS production

Production of the reactive oxygen species (ROS) NO^-^ and superoxide anion (O_2_^-^) by activated PBMC is an important innate immune response to destroy invading microbes. Cross-sectional studies assessing As influence on ROS production in children have yielded conflicting results. Analysis of 87 children in Mexico ingesting As-contaminated water showed that exposure positively associated with O_2_^-^ production by mitogen-stimulated monocytes and basal NO^-^ and O_2_^-^ levels in PBMC and monocytes [72]. This is inconsistent with an earlier study of 65 children living near a primary smelter in Mexico in which As exposure was negatively associated with NO^-^ and O_2_^-^ production by stimulated monocytes [73]. The discrepancy may be due to differences in exposure; children in the earlier study had lower urinary As levels (range 16.7-465.7 μg/g creatinine) [73] than those in the more recent study (range 12.3-1411 μg/g creatinine) [72]. Regardless of the source of variation in results, these studies suggest that As could alter circulating cells’ ability to respond to immunological challenge. For example, elevated ROS levels in un-stimulated PBMC indicate As-induced oxidative stress, concurrent with findings from copper smelter workers [27]; ROS overproduction by activated PBMC could cause oxidative damage to surrounding tissues, whereas diminished ROS production could weaken PBMC defense against pathogens.

#### Prenatal exposure

Because As readily crosses the placenta [74], it could potentially alter prenatal development. Indeed, gestational As exposure is linked to increased fetal loss and infant mortality [75,76]. However, reports on immune-related effects of prenatal exposure in newborns and infants are scarce. In a mother-child cohort study in Bangladesh (*n* = 140), maternal urinary As levels were significantly negatively correlated with child thymic index and breast milk trophic factors IL-7 (needed for thymic and T-cell development) and lactoferrin (an antioxidant and factor in innate immunity), and positively correlated with maternal morbidity and male infant RTI [77]. These findings are supported by a more recent prospective population-based cohort study of 1,552 infants born in Bangladesh, which revealed dose-dependent increases of 69% and 20% in infant lower RTI and diarrhea, respectively, related to exposure during pregnancy (maternal urinary As levels, lowest quintile < 39 μg/L; highest quintile = 262–977 μg/L) [78]. The observed increased prevalence of infant respiratory illness is consistent with As-associated adult non-malignant lung disease [35,42] and marked increased risk of such disease following early-life exposures [43]. Moreover, enhanced male infant susceptibility to RTI is consistent with increased As-related pulmonary effects in men and not in women [36].

Another study of women delivering babies in Bangladesh (*n* = 130) found that gestational As exposure induced placental inflammation (IL-1β, TNF-α and IFN-γ) via oxidative stress (8-oxoguanine), reduced placental CD3^+^ T-cell numbers, and increased umbilical cord blood IL-8, IL-1β, TNF-α and IFN-γ [79]. These findings concur with reports of elevated oxidative stress [27] and inflammation [21] in chronically-exposed adults. In a follow-up study (*n* = 44), As levels in maternal urine and placental and cord blood positively associated with cord blood 8-hydroxy-2'-deoxyguanosine and inversely associated with infant thymic function at birth, as measured by signal-joint T-cell receptor-rearrangement excision circles in cord blood mononuclear cells (CBMC) [80]. Further, prenatal As exposure was associated with down-regulated oxidative-stress defense genes, including *SOD3*, and up-regulated apoptosis-related genes in CBMC, including *CASP2*[80], the latter consistent with results from adults [22].

Overall, these data indicate that *in utero* As exposure reduces infant thymic size and function, likely through inhibiting breast milk trophic factors and/or inducing apoptosis and oxidative stress. These effects may contribute to infant immune deficiency evidenced by increased RTI prevalence. Lack of data supporting a relationship between early-life As exposure and non-pulmonary infections suggests that the developing lung is specifically targeted by As. Furthermore, given increasing evidence of As-associated adverse immune-related outcomes, it is likely that immune disruption resulting from early-life As exposure will have long-term detrimental consequences well into adulthood, as seen in increased prevalence of bronchiectasis and lung, kidney and bladder cancers.

## Experimental animal studies

###  

#### Gene expression

In various animal models, As exposure is associated with altered expression of genes involved in immune response. In lungs of mice exposed to As^III^ (< 100 ppb) for 5–6 weeks, significant changes were identified in transcripts encoding humoral immune response, antigen binding, TLRs, cytokines, cytokine receptors and genes involved in cell adhesion and migration [81,82]. Specifically, down-regulated expression of genes encoding TLR/IL1R signaling pathway, including *Il1b*, was identified [82]. In zebrafish embryos, As significantly inhibited induction of genes involved in regulating innate immune responses against viral and bacterial infection, including *il1b*[83], *tnfa*, *ifnphi1* (type1 interferon) and *mx* (interferon-inducible Mx) [84,85]. As also disrupted JAK/STAT pathway, which is critical in cytokine regulation [84]. These effects concur with epidemiological findings of As-associated decreased expression of *IL1B*[19,20] and *TNF*[22].

##### Lymphocyte subpopulations

Studies in rats [86-88], mice [89], catfish [90] and chickens [91] show that As can suppress the weight, index and/or cellularity of major immunocompetent organs, including spleen and thymus. In chronically-exposed mice, reduced CD4^+^ T-cell populations and CD4/CD8 ratio were evident, concurrent with observations in As-exposed children [67], as well as increased percentage of monocytes in splenic mononuclear cells (SMC) [92]. In catfish, As increased atypical lymphocytes and depleted lymphoid and melano-macrophage populations in head kidney (HK), a major immunocompetent organ [90,93]. Interestingly, a single intra-tracheal exposure of mice to 200 mg/kg gallium arsenide (GaAs) markedly decreased peritoneal lymphocyte counts [94] and splenic T-cell, B-cell and macrophage numbers by 58, 61 and 30%, respectively, without affecting their proportions [89].

##### Lymphocyte activation

Consistent with epidemiological observations [24-26,67], chronic As exposure inhibits mitogen-stimulated proliferation of PBMC and SMC in broiler chickens [91] and SMC in mice [92], and T-cell and B-cell proliferation in catfish spleen and HK [90,93]. Consequently, decreases have been observed in secretion of IFN-γ, IL-2, IL-6 and IL-12 in mice [92], and “IL-4-like factors” from HK T-cells in catfish [93]. An important consideration regarding animal studies is that As concentrations administered typically far exceed human exposures, which may account for differential effects observed.

##### Humoral and hypersensitivity responses

As can inhibit humoral immunity, as evidenced by suppressed *in vitro* primary and/or secondary antibody-forming cell (AFC) responses of rodent splenocytes [87,89,94-97]. IL-2 is a primary target of this inhibition in mice [98]. Further, As suppressed delayed-type hypersensitivity reaction, a response to cutaneous sensitization, in mice [94,99], rats [88,100] and chickens [91]. Compared to controls, As^III^-exposed sensitized mice demonstrated reduced lymph node cell proliferation, ear swelling, activated Langerhans cells (LC) in cervical lymph nodes, peritoneal macrophages and circulating neutrophils [99], suggesting that As inhibits LC migration to lymph nodes and subsequent T-cell activation.

##### Macrophages

Similar to humans [29], As exposure in animals suppresses macrophage production of NO^-^ and/or O_2_^-^[91,101-103], release of TNF-α [104], and phagocytosis [90,102,103]. In chronically-exposed animals, such effects may be long-term. Exposure of freshwater bivalve *L*. *marginalis* to As^III^ (1–5 ppm) for < 30 days resulted in time- and dose-dependent decreases in phagocytic efficiency and NO^-^ production in haemocytes, the major phagocytes and immune-effector cells in bivalves [102]. In a recovery assay, animals were maintained in As-free water for the same duration as exposure to evaluate immune efficiency. They demonstrated partial recovery of phagocytic potential, but inhibitory effects were still apparent; whereas NO^-^ production was restored to control levels in animals exposed to 1 ppm As^III^ for < 4 days, NO^-^ generation remained suppressed in high-dose- and long-term-exposed animals [102]. As can also induce apoptosis in macrophages, as seen in 3-fold increased DNA fragmentation in splenic macrophages from As^III^-exposed mice [103]. Further, splenic macrophages from As-treated mice demonstrated reduced adhesion and chemotactic index [105], surface I-A^k^ (MHC) class II molecule expression and antigen presentation to T-cells [106]. Similar observations have been made in humans [19,29], thus giving these results biological plausibility.

##### Immune surveillance

Altogether, these data indicate that *in vivo* As exposure can disrupt innate and humoral immunity. Studies of As influence on allogeneic immune response, i.e. rejection of MHC-mismatched allografts, suggest As disrupts the immune system’s ability to distinguish “self” from “non-self” [107,108]. In a mouse heart transplantation model, arsenic trioxide (As_2_O_3_) significantly reduced allograft rejection relative to control [107]. Similarly, As_2_O_3_ radically reduced severe symptoms of graft-versus-host disease in mice following allogeneic hematopoietic stem cell transplantation [108]. Together with reported decreased macrophage I-A^k^ class II expression and antigen presentation [106], these findings provide potential mechanisms whereby As can suppress the immune system’s ability to discriminate self from non-self antigens.

Investigation of As influence on immune system regulation revealed redistributed nTreg lymphocytes following 3-week As^III^ exposure in a rat model of multiple sclerosis, an autoimmune disease characterized by decreased nTreg cell number and function [30]. Whereas low As^III^ doses increased nTreg cell number in spleen and alleviated severity of the autoimmune condition, concentrations > 100 μg/L reduced cell numbers in blood and spleen, consistent with epidemiological findings from that study [30]. Evidently, low-dose As-mediated increased number of splenic nTreg lymphocytes inhibited generation of (auto-) immune responses, hence the beneficial effect of immunosuppression by low-dose As. Thus, two possible scenarios whereby As can interfere with self/non-self recognition exist: by i) preventing immune surveillance from recognizing “non-self” from “self”, leading to increased non-self antigen survival, as in allograft transplantation; or ii) inhibiting recognition of self antigens as “self”, which could arise from As-induced reduction of nTreg cell inhibitory activity, leading to “anti-self” antibody production indicative of autoimmune disease. Such effects would likely render the host immunocompromised and could have detrimental health consequences.

Several models indicate that As compromises the immune system’s ability to rid the host of pathogens and tumors. As-exposed mice demonstrating depressed humoral and cellular immunity displayed significantly impaired resistance against B16F10 melanoma, which resulted in 7-fold increased tumor burden [94]. As^III^-exposed zebrafish embryos and larvae exhibited significant 57- to 80-fold increased viral titers and 17- to 19-fold increased bacterial loads [85], and decreased ROS production [84,85]. Exposure-challenge studies in catfish revealed efficient pathogen colonization in distant tissues [93] and increased ulcer and septicemia susceptibility following *A*. *hydrophilia* infection [90]. Investigations in mice yield conflicting results on As influence on clearance of infection. Whereas As delayed splenic clearance of *S*. *aureus* in one study [105], in another As apparently enhanced resistance to *G*. *muris* gastrointestinal infection [109]. While reasons for these inconsistencies are unclear, it should be noted that As inhibited splenic macrophage adhesion and chemotaxis by > 50% in the earlier study [105], which could explain increased bacterial survival. However, the later study did not examine other immune functional parameters [109]; thus, it stands to reason that As concentrations used were insufficient to achieve immunosuppressive effects.

Chronic low-dose As^III^ (< 100 ppb) exposure of mice aggravated H1N1 influenza A infection severity, increasing morbidity and respiratory viral titers [110]. Early in the infection, As suppressed lymphocyte, macrophage and neutrophil migration to lungs and dendritic cell (DC) recruitment to lymph nodes, and inhibited production of 9/10 cytokines, including TNF-α and IL-1β [110], concurrent with down-regulated cytokines and adhesion- and migration-related genes in lungs of uninfected As-exposed mice [81,82]. As depressed DC migration in *in vitro* assays of bone marrow-derived DC from uninfected As-exposed relative to unexposed mice [110]. Similarly, As_2_O_3_ reduced DC density, T helper 17 (Th17) cells, which play a major role in defense against infections, and levels of the major pro-inflammatory cytokine IL-17 in airways of asthmatic mice [111,112]. Although cell counts and cytokine levels in lungs of As-exposed mice were similar to or higher than those of controls by day 7 post-infection [110], these results show that prolonged As exposure can impair immune responses against infection, and suggest that impaired response to repeated infections could promote chronic human diseases such as bronchiectasis.

## In vitro studies

###  

#### Lymphocyte activation

*In vitro* As exposure suppressed IL-2 secretion and proliferation of mitogen-stimulated lymphocytes from humans and various animal species [113-118]. A biphasic dose-dependent response was observed following As^III^ or As^V^ exposure of mitogen-stimulated human and bovine PBMC [119], demonstrating As immunosuppressive effects depend on the dose. As markedly suppressed lymphocyte secretion and/or mRNA levels of IFN-γ, IL-4 and IL-10 in different *in vitro* models [114,116,117]. As^III^ also significantly impaired differentiation of human Th17 cells by repressing their expression and release of IL-17 and decreasing expression of RORγt, which regulates IL-17, through inactivation of JNK/c-Jun pathway [120]. As^III^ further impaired Th17 cells by disrupting functions of DC, which regulate Th17 cell differentiation, via i) blocking DC differentiation through induced necrosis; ii) decreasing DC endocytotic activity; iii) repressing secretion of IL-12p70 and IL-23, two major regulators of Th17 activities, by activated DC; and iv) reducing ability of activated DC to stimulate IFN-γ and IL-17 release from Th17 cells [121].

In contrast with reduced CD4^+^ and unaltered CD8^+^ T-cell populations in children [67] and mice [92], *in vitro* As^III^ treatment of mitogen-stimulated mouse T-lymphocytes decreased CD8^+^ counts without affecting CD4^+^ counts [115]. Additionally, As^III^ inhibited early activation of mouse CD4^+^ and CD8^+^ cells, as evidenced by reduced surface CD69 expression [115], an effect that was reported at the protein level in *in vitro* As-exposed human CD4^+^ and CD8^+^ lymphocytes [122] and also at the mRNA level in lymphocytes of As-exposed humans [19].

##### Induction of apoptosis and humoral immunity

As immunosuppressive effects are further demonstrated *in vitro* by increased apoptotic rates in B-cells, T-cells, macrophages and neutrophils [64,123-127]. Prior to inducing apoptosis in TA3 mouse antigen-presenting B-cells, As^III^ inhibited activity of lysosomal protease cathepsin L, which is important in antigen processing/presentation to elicit T-cell responses [125]. This could possibly potentiate As-induced suppression of humoral immunity, for *in vitro* As exposure inhibits AFC responses of mouse splenocytes [128,129], consistent with animal studies.

##### Macrophages

As exposure disrupts monocyte/macrophage survival, development and function *in vitro*. As_2_O_3_-exposed human blood monocytes and U937 promonocytic cells underwent marked apoptosis during macrophagic differentiation, an important event in immune response, likely through inhibition of NF-κB-related survival pathways [126]. Further, As inhibited differentiation of human monocytes into macrophages, reversed macrophage-specific features, and impaired endocytosis/phagocytosis, essentially leading to macrophagic “de-differentiation” [130-134]. Interestingly, As_2_O_3_ enhanced LPS-induced macrophage TNF-α and IL-8 mRNA and secretion [130], suggesting As toxicity toward macrophages is complex, and supporting epidemiological findings of As-associated inflammation [21]. Altogether, these data support epidemiological evidence of disrupted macrophage function [29] and impaired phagocytosis/antimicrobial responses by macrophages of As-exposed mice [105].

##### Pulmonary effects

Proposed mechanisms for As-associated compromised respiratory immunity include impaired pulmonary alveolar macrophage (PAM) function, demonstrated by markedly reduced LPS-stimulated TNF-α and O_2_^-^ production [104], and decreased airway epithelial (AE) chloride secretion cystic fibrosis transmembrane conductance regulator (CFTR), an essential chloride channel for mucociliary clearance of pathogens, as seen in human CFBE41o- AE cells [135] and killifish gills [136]. In human 16HBE14o- AE cells, As restricted wound response, i.e. monolayer reformation following scraping of cultures, through MMP9 up-regulation [137] and inhibition of paracrine Ca^2+^ signaling [138], consistent with down-regulated adhesion- and migration-related genes in lungs of mice [81,82] and altered airway proteins in mice and humans [139-141]. Collectively, these data suggest As disrupts pulmonary defense through mechanisms involving i) altered PAM function, ii) decreased AE chloride secretion resulting in depressed clearance of pathogens, and iii) AE remodeling due to impaired wound response, ultimately promoting chronic lung diseases such as bronchiectasis.

## Discussion

**Figure 1:**
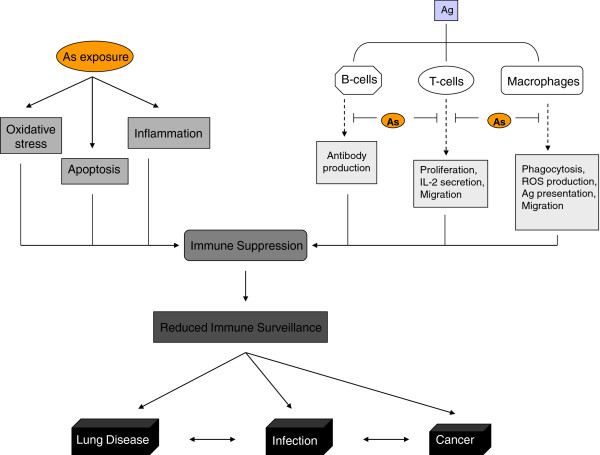
**Simplified scheme of select As-induced alterations of immune responses.** Also illustrated is how such effects might contribute to development of As-associated illnesses. Antigen (Ag) can be foreign or tumor cell.

Studies reviewed here show that As significantly impacts both innate and adaptive immune defenses. Likely mechanisms involve altered expression of key immune regulators, induced apoptosis, oxidative stress and inflammation in circulating PBMC, impaired lymphocyte activation and macrophage function, and altered cellular and humoral immunity (Table 1). Specific examples of concordance between epidemiological and experimental data are i) reduced expression of MHC class II molecules, CD69, IL-1β and TNF-α; ii) altered expression of airway adhesion- and migration-related genes/proteins; iii) decreased stimulated lymphocyte proliferation and IL-2 secretion; iv) impaired macrophage adhesion, phagocytosis and stimulated ROS production involving altered Rho A-ROCK signaling; v) induced apoptosis of PBMC; and vi) decreased stimulated ROS production by PBMC (Table 1). These effects can result in immunosuppression, as evidenced by reduced microbial clearance in animals and increased prevalence of opportunistic infections in humans, particularly RTI. Furthermore, epidemiological data suggest marked susceptibility of the lung to perturbation by As, especially during prenatal and childhood development, which results in unprecedented rates of chronic lung diseases, notably lung cancer and bronchiectasis. Experimental data suggest that such pulmonary effects could involve disrupted PAM function and airway remodeling resulting in impaired clearance of pulmonary pathogens. The pleiotropic effects of As on the immune system, including specific examples of compromised immune surveillance such as decreased rejection of MHC mismatched allografts and reduced migration of PBMC, neutrophils and DC to sites of infection in various animal models (Table 2), lend biological plausibility to increased rates of infection, cancers and other immune-related illnesses observed in As-exposed human populations, and are illustrated in Figure 1.

**Table 1:** Major findings of As-associated immune-related effects that are consistent across multiple studies

**Immune parameter**	**Major findings**	**Study model**	**Description**	**References**
Defense genes/proteins	↓ MHC class II	Humans	PBMC mRNA	[19]
		Animals	Mouse macrophage surface expression	[106]
	↓ CD69	Humans	PBMC mRNA	[19]
		Human cells	PBMC surface expression	[122]
		Animal cells	Mouse SMC surface expression	[115]
	↓ IL-1β	Humans	PBMC mRNA	[19,20]
		Animals	Mouse lung mRNA & protein	[82,110]
			Zebrafish mRNA	[83]
	↑ CD14	Humans	PBMC mRNA & surface expression	[21,30]
		Human cells	Macrophage surface expression	[130,131]
	↓ TNF-α	Humans	PBMC mRNA	[20,22]
			PBMC secretion	[26]
		Animals	Zebrafish mRNA	[84]
			Rat PAM secretion	[104]
			Mouse lung fluid protein	[110]
Inflammation	↑ Expression of inflammatory mediators	Humans	↑ PBMC *IL1B*, *IL6*, *CCL2* &*CD14* mRNA in adults	[21]
			↑ PBMC CD14 surface expression & TNF-α secretion in adults	[30]
			↑ PBMC GM-CSF secretion in children	[67]
			↑ Placental & cord blood IL-1β, TNF-α and IFN-γ in neonates	[79]
		Human cells	↑ Macrophage mRNA & secretion of TNF-α & IL-8	[130]
Lymphocyte activation	↓ Stimulated proliferation	Humans	PBMC in adults	[24-26]
			PBMC in children	[67]
		Animals	Chicken SMC & PBMC	[91]
			Mouse SMC	[92]
			Catfish SMC	[90,93]
		Human cells	PBMC	[113,114]
		Animal cells	Mouse SMC	[115,116]
			Chicken SMC	[117]
	↓ Stimulated IL-2 secretion	Humans	PBMC in adults	[26]
			PBMC in children	[67]
		Animals	Mouse SMC	[92,98]
			Catfish SMC	[93]
		Human cells	PBMC	[113,114]
		Animal cells	Mouse SMC	[115,116]
			Chicken SMC	[117]
			Harbor seal 11B7501 lymphoma B-cells	[118]
Humoral immunity	↓ AFC response to antigen	Animals	Mouse SMC	[89,94-96]
			Rat SMC	[87,97]
		Animal cells	Mouse SMC	[128,129]
Hypersensitivity reaction	↓ Response to cutaneous sensitization	Animals	↓ LC migration to lymph nodes & subsequent T-cell activation in mice	[94,99]
			Rats	[88,100]
			Chickens	[91]
Monocytes/ macrophages	↓ Number/survival	Humans	↓ Monocyte count	[28]
		Animals	↓ Mouse splenic macrophage count	[89]
			↓ Catfish HK macrophage count	[90,93]
			↑ Apoptosis of mouse splenic macrophages	[103]
		Human cells	↑ Apoptosis of blood monocytes & U937 promonocytic cells	[126]
	Impaired development	Human cells	↓ Differentiation of monocytes into macrophages	[130-133]
			Induced differentiation of macrophages into DC-like cells	[130,131]
	Diminished function	Humans	Cell rounding; ↓ adhesion/CD54 adhesion molecule, F-actin, NO^-^ production & phagocytosis; altered Rho A-ROCK signaling	[29]
		Animals	↓ Rat PAM stimulated TNF-α secretion	[104]
			↓ Mouse peritoneal macrophage NO^-^ & O_2_^-^ production	[101]
			↓ Mouse splenic macrophage adhesion, chemotactic index, phagocytosis, NO^-^ production, MHC class II surface expression & antigen presentation	[103,105,106]
			↓ Chicken SMC & PBMC NO^-^ production	[91]
			↓ Molluscan haemocyte phagocytosis & NO^-^ production	[102]
		Human cells	Cell rounding; ↓ adhesion & macrophage-specific markers; reorganized F-actin cytoskeleton resembling that of monocytes; ↑ monocytic marker CD14; ↓ endocytosis & phagocytosis via activated Rho A-ROCK signaling	[130,131]
Survival	↑ Induction of apoptosis	Humans	PBMC in adults	[30]
			↑ PBMC *BCL2L1* &*CASP2* mRNA in adults	[22]
			PBMC in children	[64,65]
			↑ CBMC *CASP2* mRNA in neonates	[80]
		Animals	Mouse splenic macrophages	[103]
		Human cells	Blood monocytes & U937 promonocytic cells	[126]
			PBMC	[64]
			B-cells, T-cells, macrophages & neutrophils	[123]
		Animal cells	Mouse TA3 antigen-presenting B-cells	[125]
			Rat T-cells	[124]
ROS production	Induced oxidative stress	Humans	↑ Serum SOD & PBMC MDA in adults	[27]
			↑ Basal PBMC/monocyte NO^-^ & O_2_^-^ in children	[72]
			↑ Placental 8-oxoguanine in neonates	[79]
			↑ Cord blood 8-hydroxy-2'-deoxyguanosine in neonates	[80]
	↓ Stimulated ROS production	Humans	↓ Macrophage NO^-^ in adults	[29]
			↓ Monocyte NO^-^ & O_2_^-^ in children	[73]
		Animals	↓ Mouse peritoneal macrophage NO^-^ & O_2_^-^	[101]
			↓ Mouse splenic macrophage NO^-^	[103,105,106]
			↓ Chicken SMC & PBMC NO^-^	[91]
			↓ Molluscan haemocyte NO^-^	[102]
			Zebrafish embryos & larvae	[84,85]
Microbial challenge	↓ Clearance of pathogens	Animals	↑ Viral & bacterial loads in zebrafish embryos and larvae	[85]
			↑ Pathogen colonization & ulcers/septicemia following bacterial infection in catfish	[90,93]
			↓ Splenic clearance of *S*. *aureus* in mice	[105]
			↑ Morbidity & respiratory viral titers following H1N1 viral infection in mice	[110]
Pulmonary health	Altered lung features	Humans	Altered airway protein expression in adults	[139,141]
		Animals	Altered mouse airway protein expression	[140,141]
			↓ Rat PAM stimulated TNF-α secretion	[104]
			↓ Mouse lung expression of genes involved in cell adhesion/migration	[81,82]
			↓ Killifish gill chloride secretion via ↑ CFTR degradation	[136]
		Human cells	↓ CFBE41o- AE cell chloride secretion via ↑ CFTR degradation	[135]
			↓ 16HBE14o- bronchial epithelial cell migration and wound repair	[137,138]
		Animal cells	↓ Rat PAM stimulated TNF-α secretion & NO^-^ & O_2_^-^ production	[104]
	↑ Risk of infection/disease	Humans	↑ RTI & tuberculosis in adults	[26,29,35]
			↓ Lung function; ↑ prevalence/mortality from lung cancer and non-malignant lung disease, including bronchitis & bronchiectasis in adults	[34,36-41]
			↑ RTI in infants	[77,78]
		Animals	↑ Morbidity & respiratory viral titers following H1N1 infection in mice	[110]

Note: all cells are primary cells unless otherwise stated. Subjects of human studies are adults unless otherwise stated.

↓ decreased; ↑ increased.

**Table 2:** Summary of specific observations of As-associated immune-related effects

**Major findings**	**Biological relevance**	**References**
↓ nTreg lymphocyte number & function in adults; redistribution in rat model of autoimmune disease	nTreg cells play critical role in immune homeostasis; alterations could affect self-recognition or influence autoimmune disease	[30]
Prenatal As exposure ↓ infant thymic size & function	Thymus is site of T-cell development; impaired function may account for ↑ prevalence of As-associated respiratory, cancer & other immune-related effects in adulthood	[77,80]
↓ CD4/CD8 T-cell ratio in children & mice	Indicator of immune suppression	[67,92]
↓ Rejection of MHC mismatched heart/bone marrow allografts in mice	↓ Immune surveillance could lead to immunocompromised state & ↓ ability to fight infection/cancer cells	[107,108]
↓ Resistance in mice against B16F10 melanoma resulted in 7-fold ↑ tumor burden	↓ Anti-tumor immunity could lead to cancer development	[94]
↓ Migration of lymphocytes, macrophages & neutrophils to lungs/DC to lymph nodes early in course of H1N1 influenza infection in mice	↓ Immune surveillance could lead to immunocompromised state & ↓ ability to fight infection/cancer cells	[110]
↓ DC density, IL-17 & Th17 cells in asthmatic mouse airways; ↓ Th17 cell differentiation & IL-17 release via disrupted JNK/c-Jun pathway & DC function	Th17 cells play a major role in defense against infection via release of major pro-inflammatory cytokine IL-17; disruption could ↓ ability to fight infection	[111,112,120,121]
↓ Urinary HBD1 peptides in men; ↓ *DEFB1* mRNA in human 293 T renal and HeLa cervical cells	HBD1 is antimicrobial peptide implicated in host anti-tumor & pulmonary immunity; its down-regulation could contribute to As-induced cancers & respiratory illnesses observed in humans	[50]

↓ decreased; ↑ increased.

## Conclusions

*In vivo* and *in vitro* studies depict As as an immunomodulator that could render the host immunocompromised. Such immune alterations could help explain increased risk of infections and several cancers observed in chronically-exposed human populations. As-mediated alterations of cellular and humoral immunity reported in animal and *in vitro* models generally agree with immunological outcomes in humans. However, more work is needed to close the gap between experimental data and risk of human immunotoxicity. Moreover, inconsistencies in epidemiological findings, possibly due to differences in dose, sampling, genetic background, and environmental/nutritional factors, indicate need for larger participant numbers and diverse ethnic populations. Due to differential effects of exposures, populations having low, intermediate and high exposure should be evaluated to better understand dose-dependent relationships. Furthermore, strong evidence for an association between developmental As exposure and elevated risk of human disease necessitates more investigations of early-life exposure outcomes. Finally, comprehensive genomic, proteomic and metabolomic profiling will be critical for identifying and validating potential molecular targets of As to monitor progression of As-associated diseases and elucidate mechanisms of As immunotoxicity.

## Abbreviations

AE: Airway epithelial; AFC: Antibody-forming cell; As: Arsenic; AsIII: Arsenite; AsV: Arsenate; As2O3: Arsenic trioxide; CBMC: Cord blood mononuclear cells; CFTR: Cystic fibrosis transmembrane conductance regulator; DC: Dendritic cells; GaAs: Gallium arsenide; GM-CSF: Granulocyte-macrophage colony stimulating factor; HBD1: Human β-defensin-1; HK: Head kidney; LC: Langerhans cells; MDA: Malondialdehyde; nTreg: Natural T regulatory; NO-: Nitric oxide anion; O2-: Superoxide anion; PAM: Pulmonary alveolar macrophages; PBMC: Peripheral blood mononuclear cells; ROS: Reactive oxygen species; RTI: Respiratory tract infections; SMC: Splenic mononuclear cells; SOD: Superoxide dismutase.

## Competing interests

The authors declare that they have no competing interests.

## Authors’ contributions

NLD, CFS and MTS conceived of the study; NLD reviewed the literature and wrote and edited the manuscript; CFS and MTS critically reviewed the manuscript. All authors read and approved the final manuscript.
